# Induced Beta Power Modulations during Isochronous Auditory Beats Reflect Intentional Anticipation before Gradual Tempo Changes

**DOI:** 10.1038/s41598-020-61044-9

**Published:** 2020-03-06

**Authors:** Emily Graber, Takako Fujioka

**Affiliations:** 1 Center for Computer Research in Music and Acoustics, Stanford University, Stanford, CA 94305 USA; 2 Wu Tsai Neurosciences Institute, Stanford University, Stanford, CA 94305 USA

**Keywords:** Cortex, Sensory processing

## Abstract

Induced beta-band power modulations in auditory and motor-related brain areas have been associated with automatic temporal processing of isochronous beats and explicit, temporally-oriented attention. Here, we investigated how explicit top-down anticipation before upcoming tempo changes, a sustained process commonly required during music performance, changed beta power modulations during listening to isochronous beats. Musicians’ electroencephalograms were recorded during the task of anticipating accelerating, decelerating, or steady beats after direction-specific visual cues. In separate behavioural testing for tempo-change onset detection, such cues were found to facilitate faster responses, thus effectively inducing high-level anticipation. In the electroencephalograms, periodic beta power reductions in a frontocentral topographic component with seed-based source contributions from auditory and sensorimotor cortices were apparent after isochronous beats with anticipation in all conditions, generally replicating patterns found previously during passive listening to isochronous beats. With anticipation before accelerations, the magnitude of the power reduction was significantly weaker than in the steady condition. Between the accelerating and decelerating conditions, no differences were found, suggesting that the observed beta patterns may represent an aspect of high-level anticipation common before both tempo changes, like increased attention. Overall, these results indicate that top-down anticipation influences ongoing auditory beat processing in beta-band networks.

## Introduction

In music, high-level anticipation is used by performers in preparing to execute subtle yet meaningful changes in the ongoing sound^1^. Such anticipation is sustained over long time periods and helps musicians coordinate spontaneous phrasing, dynamics changes, and tempo changes that are not marked in the score, but which nevertheless are required in performance^2–6^. For tempo changes, high-level anticipation may occur at the same time as other ongoing processes like those that are related to processing and predicting basic beat structure in music. Previous studies have investigated neural signatures of timing prediction and beat processing^7–11^, however their designs did not optimally allow for high-level processes, such as anticipation, to be exerted naturally as in music performance. Motivated by this, we were interested in examining how naturalistic, high-level anticipation of gradual musical tempo changes could modulate the neural activities that are ordinarily associated with beat processing. Since we do not know precisely what cognitive processes are involved in musical anticipation, this study explores the nature of anticipatory processes by examining the differences in neural activity observed when the same auditory beats are perceived with and without direction-specific top-down anticipation for gradual tempo changes. For the remainder of this study, we refer to the temporally sustained, high-level anticipation of a tempo change as *temporal anticipation*.

To see in the brain how local auditory beat processing was influenced by different types of temporal anticipation, we analysed modulations in beta power oscillations (13–30 Hz), a dynamic neural signal which has been shown to reflect time and beat processing in previous studies^7,8,12^. In general, neural oscillations are activities in various frequency ranges that occur spontaneously in the brain. When power of an oscillation falls below baseline level, it is called event-related desynchronisation (ERD), and when power is larger than baseline level, it is called event-related synchronisation (ERS)^13^. Of interest here is ERD and ERS in the beta-band range because they have been related to beat processing and timing prediction. In passively listening to auditory beats, beta ERD consistently occurred after stimuli regardless of their tempo^8^, likely representing obligatory stimulus processing^14^. Beta ERS following this ERD peaked predictively around the time of the next beat for beat intervals between 390 and 780 ms, adjusting its time course to the tempo of the stimuli^8^. For visual events occurring at slower tempos, however, no clear relationship between beta ERS rates and tempo have been found, making it necessary to investigate the previously found patterns further^15^. Nevertheless, beta power modulations for auditory stimuli at musical tempos, like those that were used in the current study, reflect the processing of beats and automatic prediction for subsequent beats according to the surrounding tempo.

Beta power modulations are also affected by high-level processes like attention, uncertainty, expectation, and imagery, all potentially involved in temporal anticipation. For attention, Todorovic *et al*.^11^ showed that beta power ERD before an actively attended tone tended to be smaller than that before an unattended tone in auditory tone-pair stimuli. When sufficient attention was paid to determine whether an auditory target was on time or delayed, beta ERS was enhanced immediately before the target^16^. Attention focused on a particular stimulus during an isochronous visual sequence enhanced beta ERS at the attended time point^17^. Beta ERS also peaked around the learned time of attended warning cues in delayed go paradigms^14,18^. Apart from the effect of attention, Tzagarakis *et al*.^19^ found that uncertainty about the spatial position of an upcoming visual target greatly reduced typical beta ERD. Similar attenuation of beta ERD was shown during a build-up of expectation for a pitch deviant in an oddball paradigm when a target was overdue to occur after standard tones^20^. Finally, imagined metric structures imposed on unaccented isochronous auditory stimuli increased beta ERD strength during the imagined accented beats^12^. Since these various high-level, endogenous processes are expressed in beta power modulations and may be expressed simultaneously as a superposition^14^, it is conceivable that temporal anticipation before tempo changes will also be expressed in beta power modulations if such anticipation involves high-level processes like those mentioned above.

Thus, we hypothesised that in anticipation of upcoming tempo changes, beta power modulations patterns may be different from those when no tempo changes are anticipated because the latter would require fewer high-level, top-down processes than the former. Further, we hypothesised that whether anticipation for different tempo changes affects ERD, ERS or both, will depend on which high-level process dominates in anticipation and when during the beat period the process takes place. Two main possibilities exist. First, if anticipation is predominantly a local predictive process where slightly early or late beats are predicted in anticipation of gradual tempo changes, then ERS rates before beats may reflect anticipation with steep/shallow rates preceding predictions for early/late beats, respectively. However, a prior study did not find an effect in the beta band when a single, specific time point was ‘targeted’ by a top-down process similar to beat prediction^7^. The second possibility is that anticipation might involve sustained attention, uncertainty, expectation, or a combination thereof rather than local expectations for an interval change; if so, sustained synchronisation of beta power might occur during multiple types of anticipation even while the stimulus stays isochronous. Such ERS would then be superimposed on the beta power modulations that are typical of passive beat processing.

With respect to neural sources, multiple brain areas contribute to the beta modulation patterns during temporal processing and could also contribute to active temporal anticipation in the current study. Using magnetoencephalography (MEG), Fujioka *et al*.^8^ found that beta modulations during passive listening to regular beats involved auditory cortices and motor-related areas such as primary sensorimotor cortex, inferior frontal gyrus, and supplementary motor areas (SMA). Morillon and Baillet^21^ showed delta-beta phase-amplitude coupling in the left sensorimotor cortex when listeners actively attended to every other tone in an auditory stream. These results are generally in line with functional magnetic resonance imaging (fMRI) studies showing that musical rhythm tracking involves simultaneous activity in bilateral auditory cortices including temporoparietal areas, bilateral premotor cortices, and SMA^22–25^. Since temporal anticipation would occur in parallel with regular beat processing, both the motor and auditory systems may be engaged as in the aforementioned studies.

**Figure 1:**
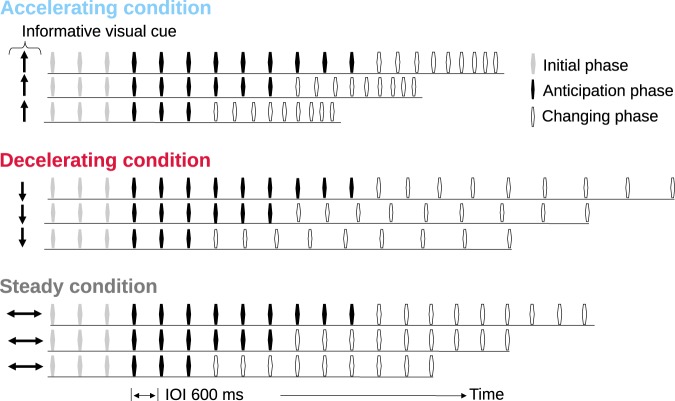
Summary of EEG stimuli. For three conditions, informative visual arrow cues indicated a tempo change direction before an auditory sequence (accelerating - up; decelerating - down; and steady - double-sided horizontal). There were three trial lengths for each condition; all sequences contained three clicks in the initial phase (indicated by the grey beat symbols) plus 3, 6, or 9 clicks in the anticipation phase (indicated by the black beat symbols) prior to any change in tempo. The last eight intervals, termed the changing phase (indicated by the open beat symbols), contained a gradual tempo change in the accelerating and decelerating conditions. In the steady condition, the last eight intervals did not contain a tempo change. Induced oscillatory responses were analysed around each beat in the anticipation phase of each trial.

In the current study, EEG was recorded from musicians as they actively anticipated and eventually heard gradual tempo changes in click sequences that contained accelerations or decelerations starting at different points in the sequences. Gradual tempo changes were used to mimic the sounds that would naturally be produced in real music performance after a temporal anticipation period. In a control condition, steady beats were anticipated for entire sequences. The direction of tempo change was visually cued prior to the onset of each sequence as illustrated in Fig. 1, but the number of steady beats before each change was not known ahead of time. Musicians were recruited for this study due to their training to anticipate gradual tempo changes while performing in an ensemble^26^, their predictive coordination abilities during expressive passages^27^, and their superior accuracy in tapping to beats in real musical excerpts compared to non-musicians^28^. In a separate behavioural session, we examined whether the visual cues facilitated anticipation by asking participants to detect acceleration or deceleration onsets with and without explicitly-cued tempo-change directions.

## Methods and Materials

The EEG methods and materials have been described in another paper which used the same EEG dataset but analyzed different aspects of the data based on a distinct set of goals^29^. Below we describe relevant information for the current study, including a separate behavioral test which was conducted after the EEG recording was completed. For further details, please refer to the previous paper.

### Participants

The current study was based on data from 20 musicians (7 female) with long-term musical training and performance experience (*M* = 22.3 years, *SD* = 5.54). All participants were right-handed. Written informed consent was obtained from the participants prior to the study. The experimental protocol was approved by the Institutional Review Board (IRB) at Stanford University. Experimental methods were carried out in accordance with the guidelines of the IRB and the Declaration of Helsinki.

### Stimuli for EEG

Figure 1 depicts the EEG trials which each contained a visual arrow cue followed by an auditory click sequence. The cues lasted 300 ms. The click sequences began 500 ms after cue offset and contained steady beats followed by a gradual tempo change. Accelerations/decelerations in tempo were cued by up/down arrows, respectively, while a horizontal, double-sided arrow was used to cue the steady condition which contained no tempo change. The click sequences started with steady inter-onset intervals (IOIs) of 600 ms for 6, 9, or 12 beats before the tempo changes began. The tempo changes lasted for eight intervals, with each interval changing by 96.85% or 103.25% of the previous interval, for accelerations and decelerations respectively. In the accelerating condition the final IOI was 464 ms, 77.33% of the initial IOI, while in the decelerating condition it was 775 ms, (1/0.7733) * 100% of the initial IOI. Each of the nine sequences (3 tempo-change conditions × 3 sequence lengths) was repeated 50 times, but presented in a pseudo-randomised order.

### Behavioural task during EEG recording

Two tasks were asked of the participants. The primary task was to actively anticipate a tempo change prior to its onset according to the informative visual cue given before the start of the sequence. This part of the task was intended to be very naturalistic for the participants who, as musicians, were used to anticipating tempo changes in ongoing music in coordination with other musicians, even without indications in the score. In giving these task instructions, it was explained that anticipating the tempo changes should be similar to anticipating upcoming tempo changes while performing ensemble music.

The secondary task took place during the changing phase of the stimuli (open beat symbols in Fig. 1) and required participants to detect any non-gradual changes in the sequences. This task was given to ensure that the participants actively, rather than passively, listened to the sequences after the primary task of anticipation was over. The non-gradual tempo changes were introduced in ten percent of trials by removing 200 ms from one interval during the changing phase. The non-gradual changes, or targets, were reported by button press. Importantly, this secondary target detection task did not contaminate the EEG with motor responses during the anticipation phase of the stimuli (black beat symbols in Fig. 1) which was the segment of interest for analysis in the current study. The EEG recorded during the secondary task was not analysed in the current study. The participants were made aware that no targets would occur during the anticipation phase of the stimuli.

### EEG recording and data analysis

The EEG was recorded in a sound-attenuated and electrically-shielded booth with 64-channel Neuroscan Quik-Cap, a SymAmpRT amplifier, and Curry 7 acquisition software (Compumedics Neuroscan Inc., El Paso, TX), at a sampling rate of 500 Hz. Offline, data were re-referenced to the common average. Doing so allows auditory activity to be expressed at central electrodes in an unbiased fashion^30,31^. Participants listened to the auditory stimuli through insert earphones (ER-1, Etymotic Research, Elk Grove Village, IL) and saw the visual cues on a monitor 1.2 metres away.

The EEG data were analysed in MATLAB (Mathworks Inc., Natick, MA) using house-made scripts with routines from the Brainstorm toolbox^32^. For preprocessing, the continuous data were bandpass filtered between 0.5 Hz and 50 Hz and down sampled to 125 samples per second. For each participant, vertical and horizontal ocular artifacts were modelled via the signal-space projection method provided in Brainstorm, and removed from the continuous data. The continuous data were parsed into epochs from −1,000–1,200 ms around individual beat onsets that occurred in the anticipation phases of the click trains (see Fig. 1). Separate analyses were carried out on other parts of the data (changing phase and inter-trial interval^29^) which are reported elsewhere. The current study focuses analysis only on the anticipation phase of the data. The epochs from the anticipation phase were baseline corrected using a time window of −100–0 ms. Note that no epochs were made from EEG segments in the initial phase (grey beat symbols in Fig. 1) or the changing phase. Any channels with peak-to-peak voltage differences exceeding ± 70 µV were rejected from individual epochs in addition to the ocular artifact rejection based on signal-space projection. This yielded 900 epochs per condition per participant.

Beta-band power modulations were computed in four steps. (1) A principal component analysis (PCA) of the grand average evoked response collapsed over all conditions and participants in the anticipation phase was performed in order to obtain spatial filters to reduce the dimensionality of the data, effectively incorporating into the filters information from across the scalp rather than from a few electrodes. Specifically, a singular value decomposition (SVD) was performed on the two-dimensional matrix of channels by samples over a one-beat interval 0–600 ms. SVD by definition breaks data into three parts: for our EEG data we obtained the eigenvectors or components describing linear combinations of channels, the time series produced by each eigenvector, and the singular values describing the contribution of each component to the overall variance of the original data. Thus, SVD yielded a set of paired spatial and temporal components, each successively accounting for less variance in the data (for more details about SVD for EEG, see Harner^33^ and Lagerlund *et al*.^34^). Only the first two spatial principal components (hereafter PCs), accounting for over 96% of the variance together, were used in the subsequent time-frequency analysis. This approach was chosen to combine redundant information in EEG channels and reduce computational burden while making use of the assumption that a majority of evoked and induced activities share neural sources^35^. Such component-based data reduction approaches with frequency and time-frequency analyses have been used widely in EEG/MEG research to investigate brain activity^36–42^.

(2) All single trial epochs were spatially filtered (projected onto the two PCs) after subtracting the individually averaged phase-locked evoked response in the corresponding conditions to isolate induced activities. (3) The time-frequency representations (TFRs) of these epochs were computed with a Morlet Wavelet decomposition with 35 logarithmically-spaced bins from 11–40.1 Hz. Signal power in each trial was calculated by multiplying each wavelet coefficient with its complex conjugate in Brainstorm routines. The TFRs were averaged separately for each participant, condition, and PC. (4) ERD and ERS in each frequency bin were computed as percent deviations from the mean power from 0–600 ms. The beta-band ERD/ERS was then computed by averaging frequency bins from 13–30 Hz and baseline correcting using a time window of −50–0 ms.

Beta ERD amplitude was the main feature of interest for statistical analyses. Amplitudes within each condition were averaged over the fixed window ± 20 ms around the grand average steady ERD peak latency and submitted to a one-way repeated measures ANOVA with the factor Condition (Accel, Steady, Decel). This was done separately for the ERD in PC1 and the ERD in PC2. Post-hoc tests were conducted by two-sided paired t-tests with Bonferroni corrections for multiple comparisons.

In order to estimate relative contributions of the neural generators underlying PC1 and PC2, dipole modelling was conducted using BESA Research 6.1 software (BESA GmbH, Grafelfing, Germany). Dipole locations were seeded based on the results of neuroimaging studies about tracking rhythmic stimuli over time. This approach has been widely used to assess the contribution of specific sources to observed event-related potentials^43–45^. By averaging coordinates reported in fMRI and positron emission tomography (PET) studies which employed tasks of attentive listening to predictable rhythmic patterns without synchronised movements or competing tasks^22–25^, five seed locations were obtained; bilateral auditory cortices extending to temporoparietal junctions and inferior parietal cortex (henceforth abbreviated as AC/TP), bilateral premotor cortices (PMC), and SMA. After the five seed locations were established, a four-shell ellipsoidal head model with the template brain in BESA was used to fit the dipole orientations to the grand average evoked response, the same data from which the two PCs were computed. The dipole orientations were determined with respect to the grand average evoked response because it most cleanly represents the neural activity related to the stimuli without bias from the different conditions. Because participants were strictly listening to the stimuli in the anticipation phase, the orientation of bilateral temporoparietal sources were fit first, followed by the bilateral PMC dipoles, and the SMA dipole. Afterward, these five sources were applied to each of the two PCs to estimate how much of their topography was accounted for by the model, as well as how much each source contributed to the topography. This estimation was conducted by simply projecting the five-dipole source model to the topographic map without changing locations or orientations.

### Post-EEG behavioural session and data analysis

After the EEG recording, the participants took part in a behavioural session. The test assessed whether the visual cues in the stimuli effectively facilitated temporal anticipation. The stimulus sequences were identical to those used in the EEG session, except that the number of isochronous beats before the changing phase was extended to include five levels: 3, 6, 9, 12, and 15 isochronous beats. These sequences were presented to participants in two separate blocks: one with cues and one without cues. Within each block, trials were pseudo-randomised, and the order in which the blocks were presented was counterbalanced across participants. In each block, the five possible trial durations were repeated three times for each anticipation condition (accelerating, decelerating, or steady). Visual cues during the cued block were always accurate and appeared exactly as they had in the EEG session.

Participants were asked to respond with a key press as soon as they detected the onset of a gradual tempo change regardless of its direction. This key press terminated the current trial immediately, and the next trial began 1.2 seconds later. If they detected no change during the trial, they responded with a key press after the trial was finished. All stimuli were controlled with a customised PsychoPy script^46^ on a Linux computer. Sounds were delivered through earbuds. The session lasted approximately 25 minutes.

Reaction time (RT) of responses to only the accelerating and decelerating trials were analysed. RTs were measured with respect to the onset of a tempo change, defined as the earliest possible moment that a change physically occurred. In accelerating trials, the onset of the first early beat was considered time zero. In decelerating trials, the onset of the first absent isochronous beat was considered time zero. Only responses occurring 200–2,800 ms after the onset of a tempo change were included in the analysis; any responses occurring more than three times the standard deviation away from individual mean RTs (within participant) were excluded from the analysis. 94.2% of the RT data was valid and examined by a three-way repeated measures ANOVA with factors Cue (cued, uncued), Condition (accelerating, decelerating), and Length (the number of beats before the changing phase; 3, 6, 9, 12, 15). This was followed by post-hoc paired t-tests with Bonferroni corrections for multiple comparisons.

## Results

### Behavioural performance

**Figure 2:**
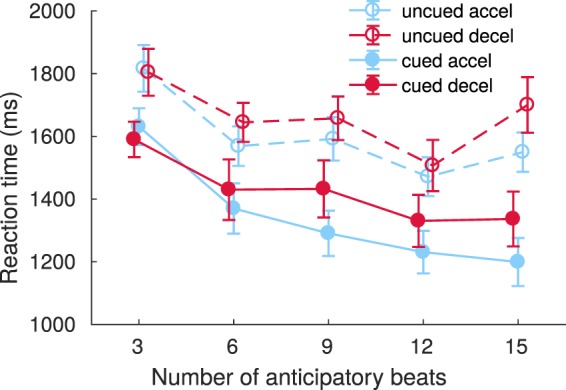
Reaction times (RTs) in the behavioural test. The RTs to detect tempo-change onsets for cued and uncued accelerations (filled and open blue symbols, respectively) and cued and uncued decelerations (filled and open red symbols) are illustrated as a function of trial length, i.e., the total number of anticipatory beats prior to tempo-change onsets. The error bars indicate standard error of the mean (SEM).

In the post-EEG behavioural test, participants detected gradual tempo-change onsets with and without informative visual cues. For RTs, the ANOVA revealed a main effect of Cue [*F*(1, 19) = 39.344, *p* < 0.0001], because RT was faster for cued (*M* = 1,384.1 ms; *SD* = 277.5 ms) compared to uncued trials (*M* = 1,631.2 ms; *SD* = 232.3 ms). The main effect of Condition was marginal [*F*(1, 19) = 3.404, *p* = 0.0807], because RT tended to be faster in accelerating trials (*M* = 1,472 ms; *SD* = 217.4 ms) than in decelerating trials (*M* = 1,543.3 ms; *SD* = 288.3 ms). Finally, the effect of the Length was significant [*F*(4, 76) = 20.862, *p* < 0.0001], because more beats before the changing phase resulted in faster RTs (Fig. 2). Post-hoc tests about the effect of Length revealed that the shortest trial length was primarily responsible for the main effect; Table 1 summarises the comparisons that were significant after Bonferroni correction. Only the interaction between Cue and Length approached significance [*F*(4, 76) = 2.261, *p* = 0.0703]. In sum, the participants responded faster when the predictive cues were available and when the trials were longer.

**Table 1:** Significant results in post-hoc comparisons of RT in different trial lengths in the behavioural test (5 levels with 3, 6, 9, 12 or 15 beats before the tempo change). *indicates *p* < .05, **indicates *p* < .01, ***indicates *p* < .001, and ****indicates *p* < .0001.

# beats before tempo change	t-statistic (df = 19)	corrected p-value (by 10)	significance level
3 vs. 6	6.202	0.0001	***
3 vs. 9	4.619	0.0018	**
3 vs. 12	9.326	0.0000001	****
3 vs. 15	5.839	0.0001	***
6 vs. 12	3.305	0.037	*

The target detection performance during the EEG recording was analysed to examine whether participants were attentive throughout the EEG trials. The *d’* sensitivity index for each participant was computed from the correct responses during target trials and false positives during non-target trials. The group mean *d’* was 4.05 (*SD* = 0.817) with a mean hit rate of 84.22% (*SD* = 23%) and false positive rate of 0.22% (*SD* = 0.25%). Differences between acceleration, deceleration, and steady targets were not assessed because the small number of targets in each condition were not consistently inserted at the same point within the changing phases. Nevertheless, the performance accuracy indicates that participants successfully attended to the stimuli during the EEG recordings.

### Principal components and source contributions

**Figure 3:**
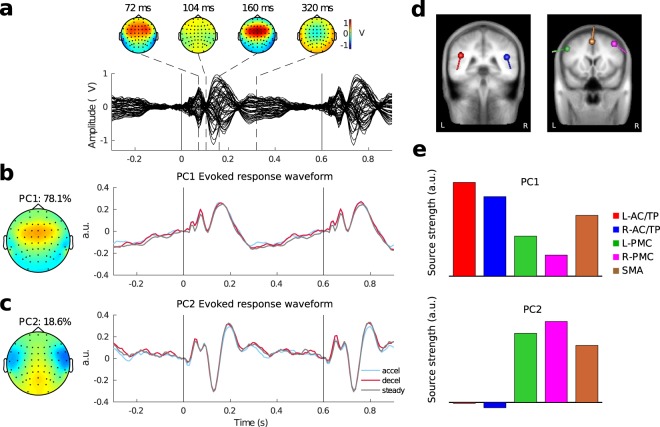
Evoked response, projections, and dipole source contributions. (**a**) Grand average evoked response across all participants and conditions from epochs around beats in the anticipation phase. Above are the topographies at latencies of 72, 104, 160, and 320 ms, falling around P1, N1, P2, and a slow response respectively. Black vertical lines mark the beat onsets at 0 and 600 ms. The top two principal components of the grand average are shown in (**b,c**). (**b**) Left: PC1 topography. Right: Evoked response projected onto PC1. (**c**) Left: PC2 topography. Right: Evoked response projected onto PC2. (**d**) Five equivalent current dipole locations in bilateral auditory cortices (red and blue symbols, indicated as L-AC/TP and R-AC/TP), bilateral premotor cortices (green and magenta symbols, indicated as L-PMC and R-PMC), and supplementary motor area (brown symbol, indicated as SMA), overlaid on the template brain MRI. (**e**) Relative amplitude contributions of the five dipoles to the PC1 and PC2 topographies, normalised.

The EEG data in channel space was parsed into epochs around the individual beats during the anticipation phase of each trial. Figure 3a shows the grand average evoked response of those epochs across all participants and all three conditions. The topographic maps above the waveform show the positive components P1 (72 ms) and P2 (160 ms) in frontocentral electrodes. The N1 response (104 ms) was less represented in the scalp topography and barely reached the baseline level because of the fast stimulus rate in the click sequences. The topographic maps of the first two PCs - from the evoked response in Fig. 3a - and their corresponding time courses are shown in Fig. 3b,c. The first PC (PC1) accounted for 78% of the variance. Its topography resembles those of the P1 and P2 responses with maximum activity at frontocentral electrodes and polarity reversals near parietal sites (Fig. 3b left). The waveform of PC1 resembles the characteristic P1-N1-P2 complex in all three conditions. The second PC (PC2) accounted for 18.6% of the variance, and its topography showed a central maximum with bilateral polarity reversals near temporal sites (Fig. 3c left). The PC2 waveform shows an N1c-like negative going peak with sustained voltages after.

For PC1 and PC2, we also determined how much the bilateral auditory cortices, bilateral premotor cortices, and SMA each contributed to the topographies using a seed-based source analysis modelled by equivalent current dipoles. The Talairach coordinates for the equivalent current dipole seed locations were as follows: bilateral AC/TP sources (Left-AC/TP: −43, −37, 22.8; Right-AC/TP: 45, −31, 19.6), bilateral PMC sources (Left-PMC: −49.4, 0.4, 37; Right-PMC: 40.6, 5.5, 46.4), and SMA (−1.7, 12.3, 50.7). Fitting the orientations of the dipoles to the grand average evoked response in Fig. 3a for a one-beat interval (0–600 ms) resulted in a model which accounted for 86.6% of the variance in that response, which is high given the wide time interval of the evoked response used for fitting the model. In fact, the model showed the strongest fit peaks of 92.13% around P1 at a latency of 52 ms and 90.05% around P2 at a latency of 162 ms. Figure 3d illustrates the locations and orientations of the five dipoles. The bar plots in Fig. 3e depict the relative contributions of the five sources to each of the two PCs. The magnitude of each source is indicated by the bar length, while its orientation (0 or 180 degrees) is indicated by the bar sign. The model explained 91.3% of the variance of the PC1 topography with the AC/TP sources contributing more than the PMC and SMA sources, though all five dipoles contributed to the fit as the bar plot shows. For PC2, the model explained 80.2% of the topography with the PMC and SMA sources contributing much more than the AC/TP sources. Notably, the PC2 topography was best explained by the available bilateral radially-oriented sources, namely the PMC sources in our model. The AC/TP sources in our model were largely tangentially-oriented in order to explain the frontocentral voltage concentration in the auditory evoked response (also the major part of the PC1 topography) and therefore contributed little to the fit. However, simply deactivating the AC/TP sources and SMA source in our model resulted in the fit of 47.9% for PC2. Therefore, we further explored the aforementioned observation that the PC2 waveform showed a N1c-like component, which could be explained by radially-oriented AC/TP sources. Indeed, re-fitting the orientation of bilateral AC/TP sources without any other sources resulted in a fit of 88.7%, better than the fit of 72.3% obtained when fitting the PMC orientations alone. This suggests that the AC/TP sources primarily contributed to the PC2 topography. Interestingly, however, keeping all radially-oriented AC/TP and PMC sources yielded the best result of 90.0%.

### Induced beta-band oscillatory activities

**Figure 4:**
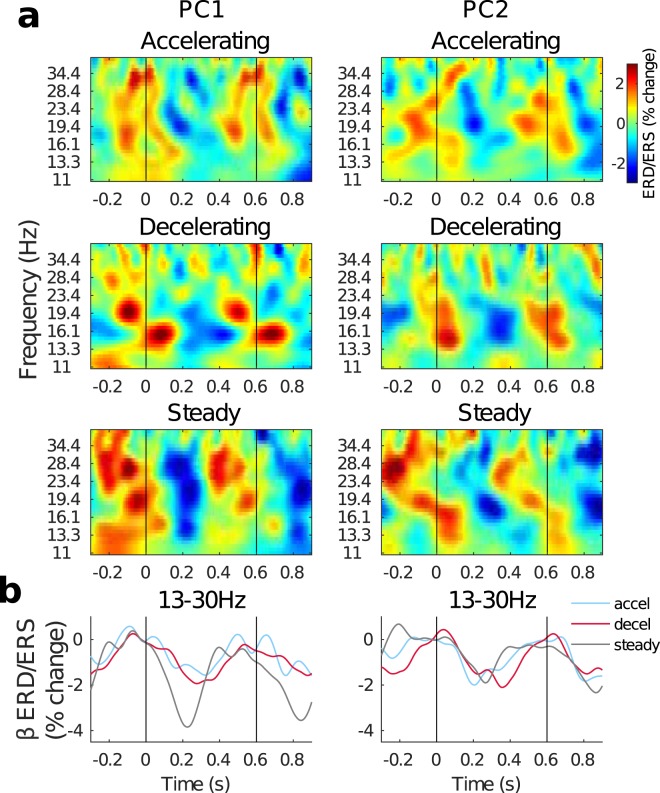
Time-frequency representations (TFRs) of the grand average induced oscillatory power activities. (**a**) TFRs for PC1 and PC2 are shown in the left and right columns, respectively. Conditions are shown in rows. Vertical black lines mark the beat onsets at 0 and 600 ms. The colours represent power as percent change relative to the mean from 0–600 ms in each frequency bin. (**b**) Beta ERD/ERS from averaging adjacent frequency bins 13–30 Hz in the TFRs in (**a**). The beta trajectories from PC1 are on the left, and those from PC2 are on the right. Vertical black lines show where beat onsets occurred. The beta-band activity in the three conditions are indicated by line colour (blue - acceleration; red - deceleration; grey - steady).

The grand average TFRs from each condition in PC1 and PC2 are illustrated in Fig. 4a. In general, the activities in the beta range have higher power (warm colours) around the beat onsets at 0 and 600 ms, and lower power between the beats (cool colours). Note that the entire beta range rather than one small sub-range is full of activity across the different conditions. Within each condition though, there may be multiple distinctive frequencies with strong activity. For the current study, in order to understand the general differences between the conditions, only broad beta-band power modulations were analysed. Averaging the activity from 13–30 Hz, and correcting the baseline from −50–0 ms resulted in beta-band power trajectories for each condition and PC, shown overlaid in Fig. 4b. In the line plots, the beta power modulation patterns described above can be seen in every condition for both components. The grand average peak latency of ERD in the steady condition (Fig. 4b grey lines), was 224 and 264 ms for PC1 and PC2, respectively. The average amplitudes of the ERD in the three conditions were computed in 40 ms time windows around the peak and compared by one-way ANOVA. For PC1, the factor Condition was significant [*F*(2, 19) = 4.252, *p* = 0.0216]. Post-hoc comparisons showed that the steady condition had the strongest ERD (lowest power), but the difference was only significant in comparison to the accelerating condition [Accel vs. Steady: *t*(19) = 3.1472, *p_corrected* = 0.0159; Decel vs. Steady: *t*(19) = 2.2532, *p_corrected* = 0.1089]. The difference between the accelerating and decelerating conditions was not significant [Accel vs. Decel: *t*(19) = 0.4806, *p_corrected* ≥ 1]. For PC2, the factor Condition was not significant [*F*(2, 19) = 0.130, *p* = 0.8787].

## Discussion

In the present study we observed that: (1) behavioural RTs to tempo-change onsets were faster when participants anticipated cued tempo changes compared to uncued changes for all trial lengths, (2) the two strongest PCs of the grand average evoked response in the anticipation phase had source contributions predominantly from bilateral temporoparietal and motor areas, and (3) induced beta-band activities in PC1 were significantly attenuated by temporal anticipation.

Our first observation was that tempo-change onset detection in post-EEG behavioural testing improved with the presence of direction-specific cues. As indicated by improved RTs, cues facilitated the detection of tempo-change onsets beyond the detection possible without cues. Such cue efficacy is in line with other studies that found reduced RTs after cuing specific information about upcoming targets^19,47^. Cues seemed to help the participants use more effective anticipation strategies regardless of the tempo-change direction and trial length, since there was no significant difference between the acceleration and deceleration conditions, nor was there a significant interaction between direction and trial length. While uncued anticipation may have involved uncertainty and attention to steady time points in order to determine if any beat was not steady, cued anticipation may have involved different processes.

RTs also improved for longer trial lengths, which is expected for two reasons. First, detecting tempo-change onsets, or what are essentially temporal errors, is easier when more contextual beats are given; many studies have shown that smaller temporal errors can be detected when occurring after longer contexts compared to shorter contexts^48–51^. Second, responding to targets after a long foreperiod also reduces RTs^52^. In general, the foreperiod effect reflects that as time goes on, certain events become more and more likely, and the increased expectation for a likely event can decrease RT^53^. In our stimuli, the participants did not know exactly when the tempo changes would start in either the cued or uncued cases, but for all the stimuli, the onsets became monotonically more likely over time and facilitated faster responses after long build-ups. It is, however, unexpected that the presence or absence of a cue did not interact significantly with variations in trial length, since uncertainty about a tempo change would decrease more strongly when cued trials last for a long time compared to uncued trials. The lack of an interaction, therefore, suggests that the anticipation process itself was not directly influenced by changes in certainty about the proximity of an upcoming tempo change. What we observed was that all trial lengths had faster RTs when the tempo-change direction was given by cue. Thus, we speculate that cued anticipation was stronger than uncued anticipation, facilitating behavioural readiness regardless of tempo-change direction. Deceleration onset detection did have marginally delayed RTs compared to acceleration onset detection, however detecting acceleration and deceleration onsets was not expected to be different because the perception of lengthened and shortened intervals around 600 ms has not been found to be biassed^54–57^. Certain exemplary ritardandi in performed music are, however, approached more smoothly than accelerandi^58^, and differences in cognitive processing could exist.

Our second observation based on EEG data was that auditory and motor sources may have contributed to anticipatory neural activities. In the neural data, a majority of the activities during temporal anticipation were accounted for by two PCs. PC1 in particular captured 78.1% of the variance in the evoked data making it informative for studying induced responses coming from the same underlying sources. PC1 exhibited a frontocentral topography similar to those in auditory evoked responses, and had contributions from auditory/temporoparietal sources as well as some contributions from motor-related sources estimated through seed-based source analysis. PC2′s central topography had more contributions from premotor cortices and SMA relative to temporoparietal sources, however exploratory analysis revealed that radially-oriented temporoparietal sources likely contributed significantly to the topography. The brain areas used in the model were documented in neuroimaging studies^22–25^ and were also consistent with beta sources in both passive and active listening found by MEG through beamforming^8,12^ and analysis of sensor topographies during music tracking^10^. These sources may constitute a complex beta network that participates in top-down temporal processes such as temporal anticipation. Since the primary goal of this analysis was only to see the relative strength of predetermined sources, further research is needed to examine how these sources interact and what functional role they play in auditory temporal processing with techniques that permit more direct source access/analysis.

Our third observation based on EEG data was that beta ERD was attenuated by temporal anticipation. In the beta-band activities, our data indicated that temporal anticipation altered beta ERD only in PC1. Given that beta-band activity from PC2 was not affected by anticipation, it is possible that the sources likely contributing to PC2 (radially-oriented bilateral auditory cortices) are not highly sensitive to top-down processes related to temporal anticipation. Nevertheless, PC1 beta ERD amplitudes in the acceleration condition were significantly reduced compared to PC1 beta ERD found in the steady condition. It is noteworthy that the ERD observed in the steady condition matches well with previous data found during passive listening to isochronous beats; maximum ERD was found around 200 ms after the beat and was likely a result of bottom-up stimulus processing^8,12,20,59^. This suggests that anticipating no change of tempo was a relatively easy task akin to passive listening. The weaker ERD for the other two conditions however, may have been due to the interference or overlap of higher-level processes required by the anticipation. Added attention given to the stimuli could likely be the cause of reduced beta ERD^11,17,60^. Such extra attention would be easy to exert in comparison to the steady condition which required little if any attention for anticipation, and both anticipated accelerations and decelerations could require added attention. Since there was little difference in RTs to acceleration and deceleration onsets in the behavioural data as well, temporal anticipation as captured by the current study seems to be similar for both types of tempo changes.

Other possible causes for reduced beta ERD include sustained effort to resist entrainment to the steady beats, similar to sustained posture or muscular force^61^ or sustained effort to resist an externally applied postural change^62^. This interpretation may not be likely however, because the motor source contributions were weaker than the auditory source contributions in PC1. Uncertainty about upcoming stimulus properties also could have resulted in diminished ERD, as shown in previous studies^19,63,64^. Due to the specific properties of our stimuli, there may have been multiple sources of uncertainty which deserve further consideration. First, as discussed above, temporal anticipation could be partially related to increasing levels of certainty over time for tempo-change onsets. However, this possibility is not fully compatible with our behavioural data which failed to show cue-specific advantages related to trial length. Admittedly, in our beta-band analyses, we did not separately examine early and late portions of the anticipation period. Therefore, it is possible that beta-band changes might be affected by the length of the anticipation period, and could be related to increasing certainty levels for a temporal change in the acceleration and deceleration conditions. Second, uncertainty about target stimuli (non-gradual tempo changes) in our EEG trials should be considered, since they, rather than tempo-change onsets, were detected in the secondary task. However, uncertainty about targets alone cannot explain the larger ERD observed in the steady condition, as all three conditions (accel, decel, and steady) would involve the same uncertainty introduced by the target detection task. In fact, beta ERD obtained in the steady condition was akin to that previously shown in passive listening paradigms^8^ involving no uncertainty processing. Third, we should consider the increased certainty level for the local beat interval in the steady condition compared to the acceleration or deceleration conditions. This could actually successfully explain the greater ERD in the steady condition, because the uncertainty of the next beat interval would reduce ERD when anticipating an acceleration or deceleration, as seen in our data. An additional remark is necessary, though, because the certainty of the tempo-change onset during anticipation, and the certainty of the beat interval timing are not independent in our paradigm; the former involves an additional process of intentional anticipation for the upcoming acceleration and deceleration as required of our musician subjects in the primary task. Lastly, because there is no tempo change in the steady condition, it is possible that uncertainty about the trial length actually increases over the course of each trial for this condition and could cancel out certainty related to the beat onset timing. In fact, no previous studies demonstrating the effects of stimulus certainty have systematically investigated different types of temporal stimulus uncertainty or their interactions with intentional processes like top-down anticipation investigate here^19,63,64^. For future studies examining musical anticipation, it will be important to dissociate contributions of intentional and incidental processing of stimulus characteristics. Finally, actively sustained expectations about upcoming stimulus timing changes may have caused reductions in beta ERD in the auditory cortex, similar to those in Chang *et al*.^20^, contributing to the overall reduction we observed in PC1. Such expectations could accompany the hypothesised increase of attention as the cause of beta ERD reduction, but they would have likely led to differential effects in the acceleration and deceleration conditions. Therefore, increased attention to the stimuli seems to best explain our findings. It should be noted however, that attention for tactile stimuli has been found to enhance beta ERD^65,66^, thus the modality of the temporal anticipation is important to consider. Altogether, the effect observed in our beta-band data is consistent with ‘high-level’ anticipation involving an increased level of sustained attention during anticipation, and possibly multiple levels of certainty about the temporal structure of the stimuli. Though differences between the acceleration and deceleration conditions were not found, the current study was unique in that it showed the effect of top-down temporal anticipation in music-like contexts, involving fine-grained temporal differences, whereas previous studies investigated coarser temporal attention^7,11,17^ or explicit auditory imagery^9,12^ rather than sustained anticipation.

A question remains regarding the degree to which imagery was involved in temporal anticipation. While anticipation could entail imagery, such imagery may enhance beta ERD rather than reduce it, as we observed. Previous work showed imagining metrical stresses on unaccented auditory beats enhanced beta ERD^12^. Also, imagined movement caused more beta ERD at the time of the movement onset^13^ compared to baseline where no movement was imagined, and demanding motor imagery caused stronger beta ERD than simple motor imagery^67^. We found other EEG and MEG studies which investigated musical and temporal imagery but only evoked responses were analysed^68–70^. It would therefore be beneficial to specifically manipulate imagery within the temporal anticipation paradigm. This could be done by providing more or less musical context via score reading to ensure the stimuli remain simple enough to result in interpretable oscillatory responses. Also unanswered is how the observed beta effect specifically relates to different types of high-level endogenous processes, although we speculate that sustained attention likely played a major role here. Manipulations of uncertainty and attention could help determine whether the hypothesised cause for the observed beta ERD reductions is supported. For example, uncertainty could be manipulated by removing the visual cues from the EEG paradigm or providing false cues. Attention could be manipulated by requiring two simultaneous and demanding tasks. The possible role of temporal prediction could be examined by manipulating the predictability of the stimulus tempo changes by contrasting step-changes (highly predictable) and gradual changes (not predictable). Additionally, analyses beyond the beta range could be carried out. In particular, alpha-band power could be affected by temporal anticipation and top-down attention. Previously, studies hypothesised that increased attention at the time of an expected visual target within a rhythmic visual sequence was responsible for alpha ERD when the target was late^71^. Also depth of alpha ERD has been related to inter-stimulus interval during predictive rhythmic visual sequences^72^. Thus, alpha and beta oscillations may both play roles in temporal anticipation.

In general, the beta power modulations we observed aligned with the timing of isochronous stimuli, supporting the working hypothesis that the brain maintains predictions based on stimulus properties^73–82^. However, our neural and behavioural results also suggest that intentional mental states like anticipation can lead to alterations of the ongoing neural processes. We demonstrated that cued anticipation afforded improved tempo-change onset detection, and the effort of anticipating tempo changes weakened beta ERD coming from a neural component that involved both auditory and motor sources. Our results suggest that multiple neural sources may be active during top-down temporal anticipation, and that increased attention is likely the cognitive function underpinning such temporal anticipation. Future work investigating temporal anticipation during actual tempo changes would be informative to further understand neural mechanisms for timing processing.

## Data Availability

The datasets obtained in the current study are available from the corresponding author on reasonable request.
